# Supplementary material for: Pathological Nodal Staging Score for Gastric Signet Ring Cell Carcinoma: A Clinical Tool of Adequate Nodal Staging
Source: Diagnostics (Basel). 2022 Sep 22;12(10):2289. doi: 10.3390/diagnostics12102289 (PMC9600920; doi:10.3390/diagnostics12102289)
Supplement: Supplementary file 1 [file diagnostics-12-02289-s001.zip › diagnostics-1879112-supplementary.pdf]

diagnostics

MDPI

Supplementary Materials

Pathological Nodal Staging Score for Gastric Signet Ring Cell Carcinoma: A Clinical Tool of Adequate Nodal Staging

Chaoran Yu †, Zhiyuan Zhou †, Bin Liu, Danhua Yao, Yuhua Huang, Pengfei Wang and Yousheng Li *

Department of General Surgery, Shanghai Ninth People's Hospital, Shanghai Jiao Tong University School of Medicine, Shanghai 200025, China
* Correspondence: guttx@hotmail.com
† These authors contributed equally to this work.

Table S1. Characterization of GSRCC patients divided into elderly (age>=70), young (age<45) and rest groups.

	Level	Rest Group (45<= Age <70)	Elder GSRCC (Age>=70)	Young GSRCC (Age<45)	*p*
n		308	182	71	
T_stage (%)	T1	57 (18.5)	41 (22.5)	18 (25.4)	0.896
	T2	34 (11.0)	19 (10.4)	7 (9.9)	
	T3	100 (32.5)	55 (30.2)	21 (29.6)	
	T4	117 (38.0)	67 (36.8)	25 (35.2)	
N_stage (%)	N0	99 (32.1)	70 (38.5)	24 (33.8)	0.775
	N1	56 (18.2)	28 (15.4)	11 (15.5)	
	N2	52 (16.9)	24 (13.2)	13 (18.3)	
	N3	101 (32.8)	60 (33.0)	23 (32.4)	
M_stage (%)	M0	265 (86.0)	167 (91.8)	58 (81.7)	0.057
	M1	43 (14.0)	15 (8.2)	13 (18.3)	
Sex (%)	Male	142 (46.1)	94 (51.6)	51 (71.8)	<0.001
	Female	166 (53.9)	88 (48.4)	20 (28.2)	
Tumorsize (%)	<3cm	66 (21.4)	47 (25.8)	18 (25.4)	0.38
	3cm<= <6cm	97 (31.5)	55 (30.2)	16 (22.5)	
	>=6cm	84 (27.3)	55 (30.2)	25 (35.2)	
	unknown	61 (19.8)	25 (13.7)	12 (16.9)	
Surgery_status (%)	Surgery not performed	11 (3.6)	3 (1.6)	1 (1.4)	0.345
	Surgery performed	297 (96.4)	179 (98.4)	70 (98.6)	
Bone_M (%)	No	306 (99.4)	181 (99.5)	70 (98.6)	0.118
	Unknown	0 (0.0)	0 (0.0)	1 (1.4)	
	Yes	2 (0.6)	1 (0.5)	0 (0.0)	
Brain_M (%)	No	308 (100.0)	181 (99.5)	70 (98.6)	0.173
	Unknown	0 (0.0)	1 (0.5)	1 (1.4)	
Liver_M (%)	No	307 (99.7)	182 (100.0)	70 (98.6)	0.102
	Unknown	0 (0.0)	0 (0.0)	1 (1.4)	
	Yes	1 (0.3)	0 (0.0)	0 (0.0)	
Lung_M (%)	No	307 (99.7)	182 (100.0)	70 (98.6)	0.102
	Unknown	0 (0.0)	0 (0.0)	1 (1.4)	

	Yes	1 (0.3)	0 (0.0)	0 (0.0)	
Tumor_position (%)	C16.0-Cardia, NOS	44 (14.3)	31 (17.0)	7 (9.9)	0.539
	C16.1-Fundus of stomach	10 (3.2)	5 (2.7)	5 (7.0)	
	C16.2-Body of stomach	28 (9.1)	16 (8.8)	8 (11.3)	
	C16.3-Gastric antrum	99 (32.1)	58 (31.9)	16 (22.5)	
	C16.4-Pylorus	12 (3.9)	6 (3.3)	0 (0.0)	
	C16.5-Lesser curvature of stomach NOS	34 (11.0)	14 (7.7)	10 (14.1)	
	C16.6-Greater curvature of stomach NOS	17 (5.5)	10 (5.5)	3 (4.2)	
	C16.8-Overlapping lesion of stomach	37 (12.0)	24 (13.2)	13 (18.3)	
	C16.9-Stomach, NOS	27 (8.8)	18 (9.9)	9 (12.7)	

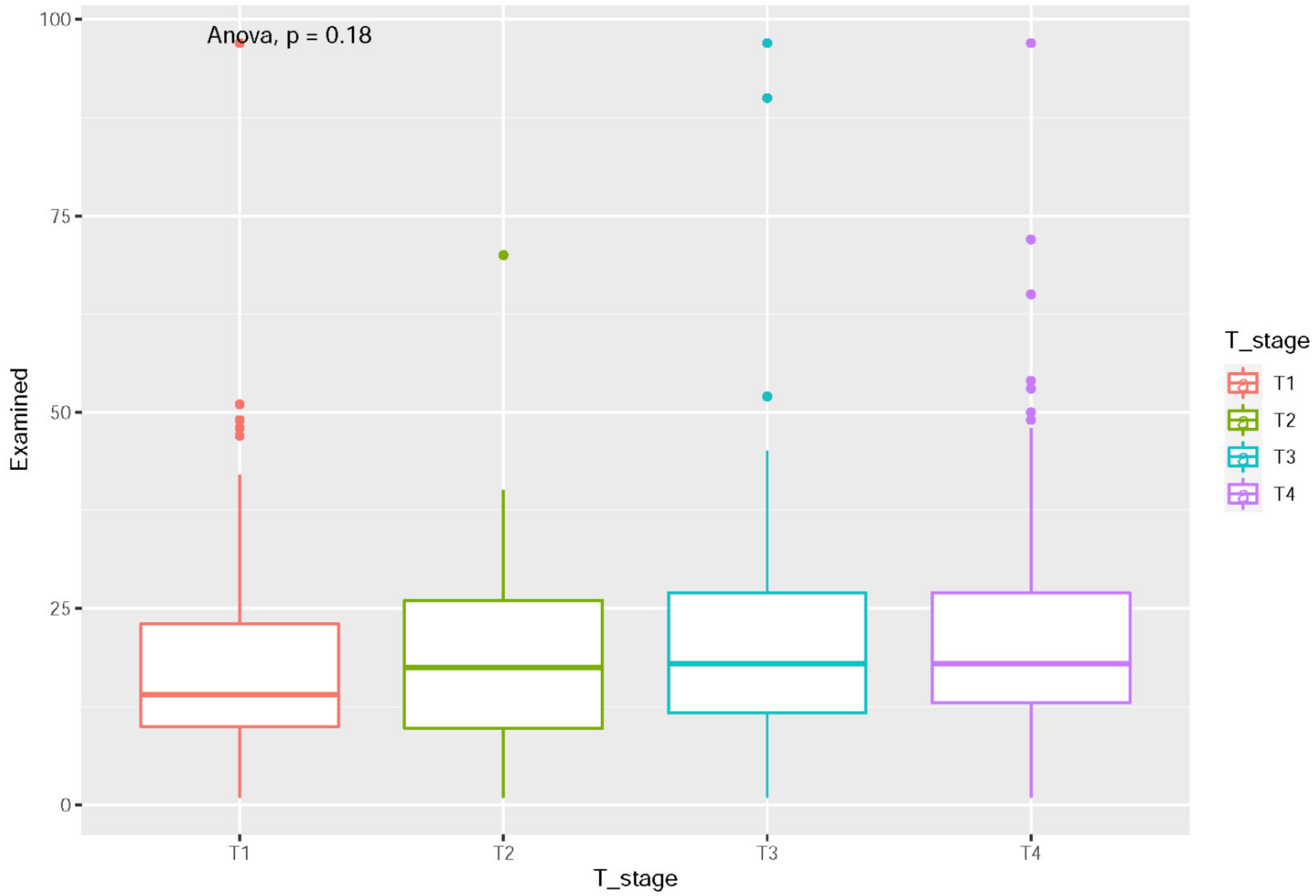

Figure S1. Boxplot of number of examined lymph nodes across various T stage.

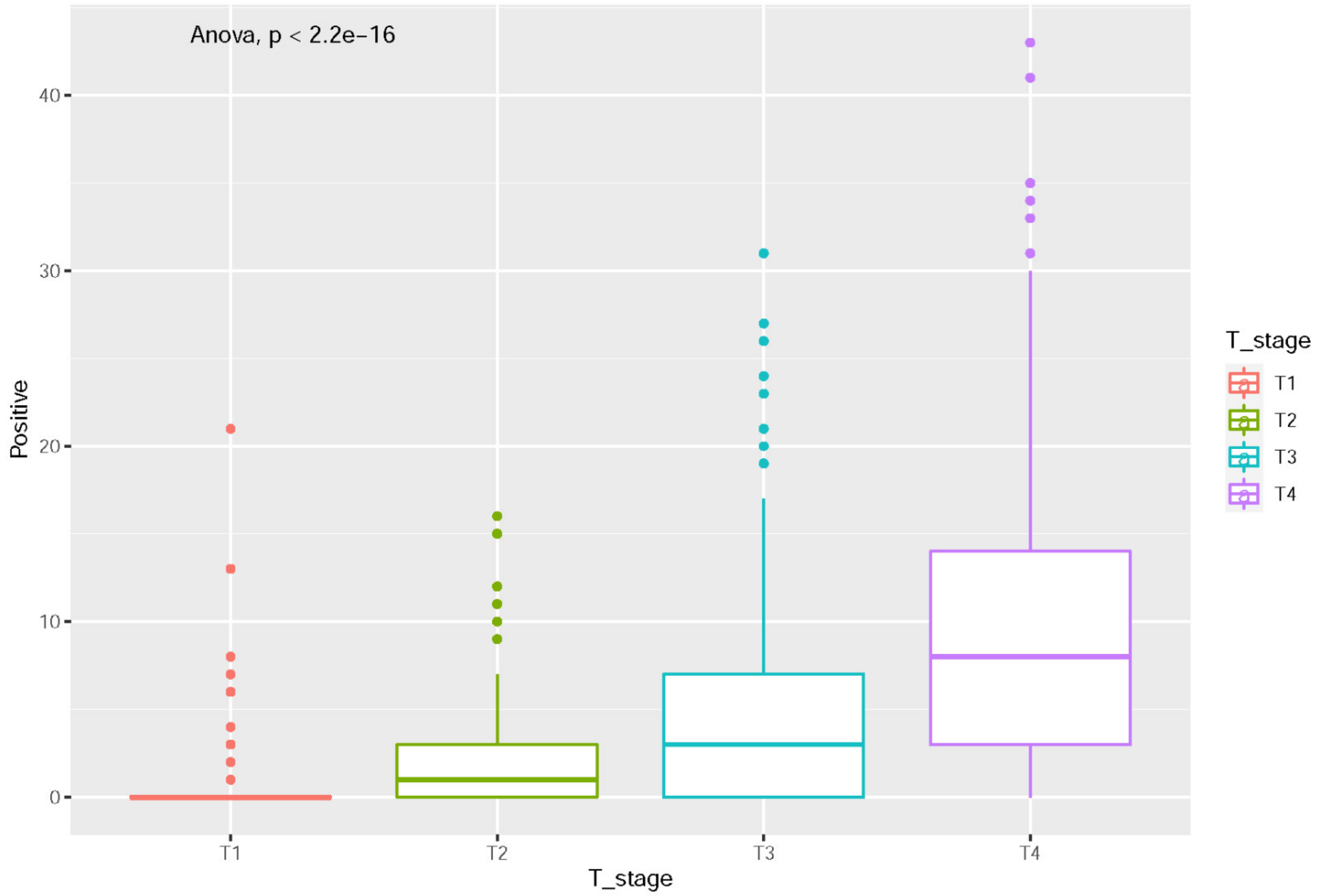

Figure S2. Boxplot of number of positive lymph nodes across various T stage.
